# Lactobacillaceae differentially impact butyrate-producing gut microbiota to drive CNS autoimmunity

**DOI:** 10.1080/19490976.2024.2418415

**Published:** 2024-10-27

**Authors:** Theresa L. Montgomery, Lucinda C. Toppen, Korin Eckstrom, Eamonn R. Heney, Josephine J. Kennedy, Matthew J. Scarborough, Dimitry N. Krementsov

**Affiliations:** a Department of Biomedical and Health Sciences, University of Vermont, Burlington, VT, USA; b Department of Civil and Environmental Engineering, University of Vermont, Burlington, VT, USA; c Department of Microbiology and Molecular Genetics, University of Vermont, Burlington, VT, USA; d Clinical Pathology, University of Vermont Medical Center, Burlington, VT, USA; e Gund Institute for Environment, University of Vermont, Burlington, VT, USA

**Keywords:** Microbiome, multiple sclerosis, SCFA, butyrate, ASF, bacteriocin

## Abstract

**Background:**

Short-chain fatty acids (SCFAs), produced by the gut microbiota, are thought to exert an anti-inflammatory effect on the host immune system. The levels of SCFAs and abundance of the microbiota that produce them are depleted in multiple sclerosis (MS), an autoimmune disease of the central nervous system (CNS). The mechanisms leading to this depletion are unknown. Using experimental autoimmune encephalomyelitis (EAE) as a model for MS, we have previously shown that gut microbiomes divergent in their abundance of specific commensal *Lactobacillaceae, Limosilactobacillus reuteri* (*L. reuteri*) and *Ligilactobacillus murinus* (*L. murinus*), differentially impact CNS autoimmunity. To determine the underlying mechanisms, we employed colonization by *L. reuteri* and *L. murinus* in disparate gut microbiome configurations *in vivo* and *in vitro*, profiling their impact on gut microbiome composition and metabolism, coupled with modulation of dietary fiber in the EAE model.

**Results:**

We show that stable colonization by *L. reuteri*, but not *L. murinus*, exacerbates EAE, in conjunction with a significant remodeling of gut microbiome composition, depleting SCFA-producing microbiota, including *Lachnospiraceae*, *Prevotellaceae*, and *Bifidobacterium*, with a net decrease in bacterial metabolic pathways involved in butyrate production. In a minimal microbiome culture model *in vitro*, *L. reuteri* directly inhibited SCFA-producer growth and depleted butyrate. Genomic analysis of *L. reuteri* isolates revealed an enrichment in bacteriocins with known antimicrobial activity against SCFA-producing microbiota. Functionally, provision of excess dietary fiber, as the prebiotic substrate for SCFA production, elevated SCFA levels and abrogated the ability of *L. reuteri* to exacerbate EAE.

**Conclustions:**

Our data highlight a potential mechanism for reduced SCFAs and their producers in MS through depletion by other members of the gut microbiome, demonstrating that interactions between microbiota can impact CNS autoimmunity in a diet-dependent manner. These data suggest that therapeutic restoration of SCFA levels in MS may require not only dietary intervention, but also modulation of the gut microbiome.

## Graphical Abstract

**Figure:**
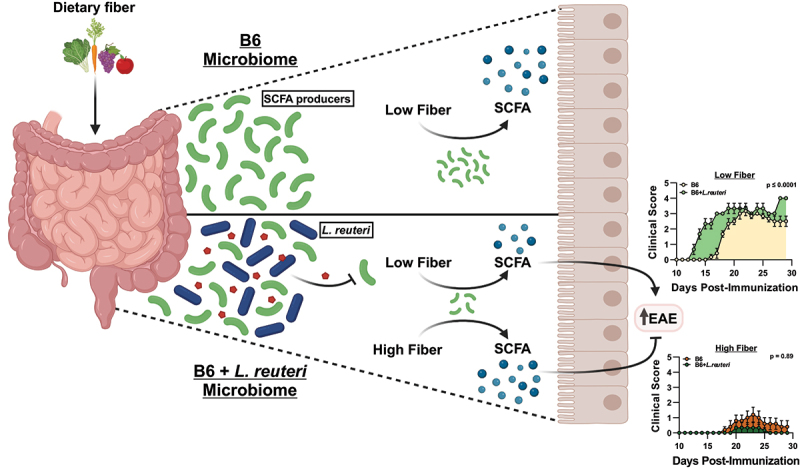

## Introduction

Multiple sclerosis (MS) is a chronic autoimmune disease of the central nervous system (CNS) and the most common cause of nontraumatic neurological disability in young adults, affecting an estimated 2.8 million people worldwide.^1–4^ Pathological changes in MS are mediated by infiltration of autoreactive T cells into the CNS, initiating an inflammatory cascade causing inflammation, demyelination, gliosis, destruction of the myelin sheath, and oligodendrocyte loss.^3–5^ Neurological symptoms and disability are a direct consequence of the immunological and pathological changes inherent to MS, including sensory, motor, psychiatric, and visual disturbances.^6–8^

The etiology of MS is complex and multifactorial, stemming from both genetic and environmental risk factors.^9–11^ In a genetically susceptible individual, environmental risk factors account for approximately 70% of disease risk and include EBV infection, diminished vitamin D3 levels, reduced UV radiation exposure, cigarette smoking, obesity, and diet.^9,12^ The gut microbiome represents a more recently identified environmental risk factor for CNS autoimmunity. Functionally, the gut microbiota can impact both the innate and adaptive immune system in the CNS and/or periphery through direct interactions with host immune cell subsets to alter their development, maintenance, and function or through production of immunomodulatory metabolites including amino acid derivates, bile acids, and short-chain fatty acids (SCFAs).^13–32^ SCFAs enter host systemic circulation by passively diffusing across cellular membranes and the BBB to exert far reaching effects on host physiology.^33^ Functionally, SCFAs are considered broadly beneficial, aiding in maintenance of barrier integrity and exerting an anti-inflammatory effect by influencing regulatory T cell differentiation and inhibiting inflammatory function of immune cell subsets in the periphery and CNS and epithelial cells of the BBB.^20,34–43^

Multiple case-controlled studies have characterized the compositional changes in the gut microbiota in people with multiple sclerosis (pwMS), including altered abundance of *Akkermansia muciniphila*, *Faecalibacterium*, *Prevotella*, *Blautia*, and SCFA-producing microbiota, including *Butyricimonas, Bacteroides, Lachnospiraceae, and Eubacterium* species.^44–52^ Recent work from our lab has also shown that depletion of SCFA producers is predictive of MS progression.^53^ Moreover, metabolomic evidence from pwMS suggests a reduction in SCFAs, including acetate, propionate, butyrate, isobutyrate, valerate, and isovalerate in serum, plasma, and/or fecal samples.^13–17,21,54–56^ Reduced SCFA levels were also found to correlate with increased intestinal permeability, inflammatory immunological responses, and/or disease worsening in MS.^15,17,54^ Moreover, acetate, propionate, butyrate, mixtures of SCFAs, and high fiber diets, all have been shown to diminish pathogenesis of experimental autoimmune encephalomyelitis (EAE), the primary autoimmune model of MS, and exert an anti-inflammatory role.^13,16,18–20,57^ However, understanding the precise underlying drivers of reduced SCFA-producing gut microbiota in pwMS, including relevant interactions between constituents of the microbiota, their metabolites, and ultimately the consequences to the host, continues to be challenging.

SCFAs are produced by the gut microbiota through fermentation of host dietary fibers and non-digestible starches within the lumen of the gastrointestinal (GI) tract.^58^ Butyrate production occurs through four avenues, including the acetyl-CoA, glutarate, 4-aminobutyrate, and lysine pathways all of which merge in the conversion of crotonyl-CoA to butyryl-CoA, as an energy conserving step for the microbiota.^59,60^ Terminal production of butyrate utilizes pathway-specific enzymes including butyryl-CoA:4-hydroxybutyrate CoA transferase (4Hbt) in the 4-amino-butyrate pathway, butyryl-CoA:acetoacetate CoA transferase (Ato) in the lysine pathway and either butyryl-CoA:acetate CoA transferase (But) or butyrate kinase (Buk) in the glutarate and acetyl-Co pathways.^60^ Butyrate-oxidizing bacteria directly consume butyrate as an energy source, in a catabolic pathway consisting of three essential enzymes converting butyrate to 3-hydroxybutyryl-CoA, ultimately yielding acetate.^61^ While less studied than their butyrate-producing counterparts, consumers can directly limit availability of butyrate to the host.

Interactions between bacterial members of the gut microbiota consist of both cooperative and competitive networks, creating positive and negative feedback loops within microbial communities dictating community-wide functional activity and interactions with the host.^62,63^ Positive interactions consist of signaling molecules involved in communication among the microbiota, cross-feeding of secreted metabolites, and detoxification of the local environment.^64^ Negative interactions can arise from competition for resources or space and consistent of production of toxic waste products and small antimicrobial peptides targeting closely related taxa, or so-called bacteriocins.^65^ Bacteriocins have long been studied for their application in food preservation, but their impact on underlying gut microbiome stability and function in the context of human health and disease has not been extensively explored.^66,67^

In our previous work, in a series of gut microbiome transplantation and commensal colonization studies, we found that divergent gut microbiome compositions naturally present in C57BL/6J (B6) and wild-derived and genetically divergent PWD/PhJ (PWD) mice differentially impact disease pathogenesis in the EAE model.^68,69^ When transplanted into germ-free (GF) B6 mice, PWD gut microbiota exacerbated disease as compared to B6 transplant recipients. Using 16S sequencing to profile the gut microbiota, we found that the B6 and PWD gut microbiomes were distinguished by the abundance of signature *Lactobacillaceae* members. While PWD mice were colonized with a high abundance of *Limosilactobacillus reuteri* (*L. reuteri*) with an absence of *Ligilactobacillus murinus* (*L. murinus*), in B6 mice, the inverse relationship of these bacterial species was observed. Furthermore, colonization of *L. reuteri* (isolated directly from the PWD microbiota) into mice harboring B6 gut microbiota, was sufficient to exacerbate EAE pathogenesis, enhancing infiltration of T cell subsets and inflammatory cytokine production in the CNS.^69^ These data suggest a divergent impact of *Lactobacillaceae* on CNS autoimmunity, which could be mediated by indirect effects on the global composition of the gut microbiota.

We therefore asked whether *L. reuteri* colonization drives global remodeling of gut microbiome composition to exacerbate CNS autoimmunity, while *L. murinus* colonization, does not. Using a series of colonization studies with *L. reuteri* and *L. murinus*, we demonstrate that *L. reuteri* exerts a more profound impact on the gut microbiota, depleting beneficial SCFA-producing gut microbiota, including *Lachnospiraceae*, *Prevotellaceae*, and *Bifidobacterium*. Further, the genome of *L. reuteri*, as compared to *L. murinus*, was enriched in bacteriocins with known activity against SCFA producers, and introduction of *L. reuteri* into minimal microbiome configurations *in vitro* was sufficient to reduce the abundance of SCFA producers and their resulting SCFA metabolites, including butyrate and isovalerate. Finally, in the EAE model, provision of excess dietary fiber, as the prebiotic substrate microbiota utilize to produce SCFAs, increased fecal butyrate levels and abrogated the ability of *L. reuteri* to exacerbate EAE, suggesting that the effect of *L. reuteri* on EAE pathogenesis can be mitigated with provision of excess pre-biotic substrate to SCFA-producing gut microbiota.

## Materials and methods

### EAE induction and evaluation

EAE was induced in 8-week-old offspring from ex-germ-free (GF)-B6 transplant recipients (B6: *n* = 9, B6 + *L. reuteri*: *n* = 6, PWD: *n* = 15, PWD + *L. murinus*: *n* = 16) using the 2×MOG35-55/CFA protocol as previously described.^68^ Mice were injected subcutaneously with 0.1 mg of myelin oligodendrocyte glycoprotein peptide 35–55 (MOG35-55) (New England Peptide, Inc. MA, USA) emulsified in PBS and 50% complete Freund’s adjuvant (CFA; Sigma, USA) supplemented with 4 mg/ml H37Ra (Difco, USA) on day 0 (lower flank) and day 7 (lower flank) at 50 µl per flank. Evaluation of disease severity started on day 10 as follows: 1 – loss of tail tone, 2 – loss of tail tone, weakened hind limbs, 3 – hind limb paralysis, 4 – hind limb paralysis and incontinence, 5 – quadriplegia or death. Significant difference in disease course was determined by Friedman’s non-parametric two-way ANOVA using the treatment by time interaction as previously described.^70^ Cumulative disease score (CDS) was calculated by taking the summation of daily scores over the 30-day disease course with significance calculated using Kruskal–Wallis with Dunn’s post-hoc comparisons.^71,72^ Disease severity was calculated using the area under the curve (AUC) of each representative EAE course by graphing total peak area and standard area of the mean, and calculating significance with a one-way ANOVA and Šidák correction for multiple comparisons.

Breeding pairs and experimental offspring were provided a diet containing 5% crude fiber, Prolab Isopro RMH 3000 cat# 5P75. For dietary intervention studies, mice were randomized by microbiome (PWD: *n* = 13, B6: *n* = 15, B6 + *L. reuteri*: *n* = 20) to 2–3 mice per cage receiving either a low (TD00102, 0% fermentable fiber) or high (TD200242, 20% inulin, 10% pectin) fiber diet prior to EAE induction at 8–12 weeks of age. Diets were vacuum packed, irradiated, stored at 4°C until use, refreshed weekly, and provided on the cage floor to mice displaying hind limb paralysis (at a clinical score of 3 or above) along with Napa nectar.

### Lactobacillus isolation and cultivation

*Lactobacillaceae* were isolated as previously described.^69^ Briefly, *Limosilactobacillus reuteri* (*L. reuteri*) and *Ligilactobacillus murinus* (*L. murinus*) were isolated from cecal contents of a PWD and B6 mouse, respectively. Cecal contents were suspended in 50 ml DeMan, Rogosa and Sharpe (MRS; Thermofisher, Inc, USA) medium supplemented with 0.25 g/L L-cysteine (Sigma, USA), 20 µg/ml vancomycin (Sigma, USA) adjusted to pH 5 and incubated anaerobically overnight at 37°C, with 200 rpm shaking, plated onto agar medium of the same formulation and incubated anaerobically overnight at 37°C. Isolated colonies were cultured overnight in MRS medium, cryopreserved, and screened by qPCR using species-specific primers.^69^ Positive isolates for each respective *Lactobacillaceae* member were cultured in vancomycin-free medium followed by repeat screening by qPCR. For colonization studies, gavage stocks were prepared using three isolates per species grown to log-phase, adjusted to OD600 = 0.5 with fresh medium. Colony forming units (CFU) of gavage stocks were quantified by standard serial dilution and plating.

### Microbiome transplantation

Gut microbiome transplantation was performed as a one-time inoculation to parental GF breeding pairs to generate experimental offspring wherein specific gut microbiome configurations were vertically transmitted as previously described.^68,69^ Briefly, the total cecal contents from B6 or PWD mice were isolated under anaerobic conditions and cryopreserved in 20% glycerol in Hungate tubes at −80°C. GF 4–5-week-old C57BL/6J mice were purchased from the National Gnotobiotic Rodent Resource Center at University of North Carolina School of Medicine (Chapel Hill, NC, USA). GF mice received in sterile crates were randomized to breeding pairs by date of birth and inoculated by gastric gavage with 100 µl of cryopreserved PWD (naturally *L. murinus* absent and *L. reuteri* present) or B6 (naturally *L. reuteri* absent and *L. murinus* present) cecal content with or without supplementation of 100 µl 10^9^ CFU *L. reuteri* or *L. murinus* under a laminar flow hood. All animals were maintained under barrier conditions, provided sterilized food, water, and caging. Handling was minimized to avoid cross-contamination between distinct microbiomes, to prevent introduction of additional environmental microbiota, and followed a strict handling order with decontamination of equipment and supplies between each group as follows: 1) PWD, 2) PWD + *L. murinus*, 3) B6, 4) B6 + *L. reuteri*. Measures taken to preserve experimental microbiome integrity have been described previously.^68^ All experimental procedures were approved by the Animal Care and Use Committee at the University of Vermont.

### Microbial DNA isolation and 16S sequencing

Fecal samples were collected (D0: B6: *n* = 9, B6 + *L. reuteri*: *n* = 10, PWD: *n* = 8, PWD + *L. murinus*: *n* = 8; D30: B6: *n* = 9, B6 + *L. reuteri*: *n* = 10, PWD: *n* = 8, PWD + *L. murinus*: *n* = 6) as previously described.^68,69^ Briefly, individual mice were placed in sterile unbedded cages and allowed to defecate, fecal samples collected, and stored at −80°C until extraction. DNA was extracted using the QIAamp PowerFecal Pro DNA extraction kit (Qiagen, USA) and assessed via Nanodrop, Qubit, and agarose gel electrophoresis. 16S rRNA amplicon sequencing was performed at the University of Illinois Carver Biotechnology Center (CBC). 16S amplicon libraries were generated using the Fluidigm Access Array (Fluidigm, USA) amplification. The following primer sequences were used to generate V3-V4 amplicons, following primers: V3_F357_N 5’-CCTACGGGNGGCWGCAG-3’ and V4_R805 5’-GACTACHVGGGTATCTAATCC-3’. The final Fluidigm pools were transferred from the Functional Genomics laboratory to the DNA Services laboratory of the CBC at the University of Illinois at Urbana – Champaign. The final pools were quantitated using Qubit (Life Technologies, Grand Island, NY) and qPCR on a BioRad CFX Connect Real-Time System (Bio-Rad Laboratories, Inc. CA). The pool was mixed evenly based on the qPCR values, denatured, spiked with 20% indexed PhiX control library, and loaded onto a MiSeq V2 flowcell at a concentration of 8pM for cluster formation and sequencing at a read length of 250nt.

### 16S data preprocessing and taxonomic assignment

Raw sequencing reads were analyzed using *DADA2* (version 1.24.0) in R (version 4.2.1).^73,74^ PhiX reads were removed, reads trimmed at 240nt, denoised at a maximum number of errors (maxEE) of 1 and a quality score of 11, followed by standard error learning and chimera removal using the consensus method. Taxonomy was assigned using the SILVA database (version 138.1).^75^ A phylogenetic tree was constructed using *DECIPHER* (version 2.26.0) to align unique sequences and using *phangorn* (version 2.11.1) to fit a maximum likelihood tree using neighbor-joining optimized with nearest-neighbor interchange in a generalized time reversible model and rooted using midpoint rooting.^76–78^ Downstream analysis was conducted with *phyloseq* (version 1.40.0).^79^ Reads that did not align to the bacterial Kingdom, *Cyanobacteria as* likely contaminants, and phyla falling below a mean prevalence of 1 (*Patescibacteria*) were removed.

### Diversity analysis

Alpha diversity (Simpson) was calculated using the estimate_richness function in *phyloseq* (version 1.40.0) statistical difference of the mean determined by Wilcoxon rank sum non-parametric testing.^80^ Beta diversity as measured by Bray – Curtis dissimilarity was calculated using the distance function in *phyloseq* (version 1.40.0) and represented as a PCoA with a statistical analysis of group differences using multivariate analysis of variance (adonis2) using the vegan package (version 2.6–4).^81,82^

### Taxonomic bar graphs and differential abundance testing

Bar graphs are the top 11 most abundant taxa, excluding ASVs that do not assign to the rank of genus. For differential abundance testing, taxa were agglomerated at each rank using the tax_glom function in phyloseq for statistical testing using *DESeq2*.^83,84^ Fold-change at the ASV level represents a taxonomic ‘best hit’ which was assigned using the format_to_besthit function in *microbiomeutilities* (version 1.00.16). For taxonomic association trees, data was normalized using total sum scaling and filtered at a minimum prevalence of 0.05 prior to linear regression modeling of differentially abundant taxa at p_adj_ . <0.05 and plotting at the rank of genus and taxonomic best hit using the taxatree_plots function in *microViz* (version 0.10.6).^85,86^ For differential heat trees comparing taxa across time, taxa were pruned at a total sum abundance across all samples of greater than 500, transformed into per sample proportions and a Wilcoxon rank sum non-parametric test applied to assess differences in the median abundance of each taxa between microbiomes and/or over time. The resulting heat tree matrix was plotted where node color represents the log2 media proportion of differentially abundant genera at p_adj_ <0.05 in a Davidson-Harel layout with node localization using Reingold-Tilford.^87^

### Pathway enrichment analysis

The metagenomic content of differentially abundant ASVs between the B6 and B6 + *L. reuteri* or the PWD and PWD + *L. murinus* gut microbiomes at day 30 was inferred using *PICRUSt2*.^88,89^ Differential abundance of unstratified enzyme commission (EC) numbers and MetaCyc pathways were analyzed using *DESeq2*.^83,84^ Stratified ASV contribution to individual ECs were generated using *PICRUSt2* to evaluate contribution to butyrate production and consumption. Butyrate producers were defined as bacteria conserving *any* of the following terminal enzymes in butyrate-producing pathways: butyryl-CoA:4-hydroxybutyrate CoA transferase (4Hbt; EC:2.8.3.-), butyryl-CoA:acetoacetate CoA transferase (Ato; EC:2.8.3.9), butyryl-CoA: acetate CoA transferase (But; EC:2.8.3.8) or butyrate kinase (Buk; EC:2.7.2.7).^60^ Butyrate consumers were defined as bacteria conserving *each* of the following enzymes: acetate CoA-transferase (But; EC:2.8.3.8), acyl-CoA dehydrogenase (Acd; EC:1.3.8.1) and either crotonobetainyl-CoA hydratase (Crt; EC:4.2.1.149) or enoyl-CoA hydratase (Ech; EC:4.2.1.17).^61,90^ Abundance of butyrate producers and consumers was plotted both in total ASV and in differentially abundant ASVs between the B6 and B6 + *L. reuteri* gut microbiomes with statistical significance determined using Wilcoxon rank sum non-parametric testing between groups. Differential heat trees of butyrate producing and consuming microbiota were generated by subsetting total microbiota to include species conserving enzymes involved in butyrate metabolism described above and pruning resulting taxa to remove low abundance reads for analysis and plotting consistent with heat trees of the total microbiota.

### Bacterial competition assay

*In vitro* culture of the modified four-member Altered Schaedler’s Flora (ASF4) was conducted as follows: ASF members including ASF356 (*Clostridium sp*.), ASF500 (*Pseudoflavonifractor sp*.), ASF502 (*Clostridium sp*.) and ASF519 (*Parabacteroides goldsteinii*), with or without *L. reuteri* were grown in brain heart infusion (BHI; Sigma, USA) supplemented with 5% each of new born calf (Thermofisher, Inc, USA), horse (Thermofisher, Inc, USA), and sheep serum (Sigma, USA), 5 g/L yeast extract (Sigma, USA), 0.2 mL of vitamin K1 solution (Sigma, USA; 0.5% vitamin K1 dissolved in 99.5% ethanol), 0.5 mL/L of hemin solution (Sigma, USA; 0.5 g/L dissolved in 1% NaOH, 99% deionized water), 0.5 g/L cysteine (Sigma, USA), and 1 mm tryptophan in an anaerobic chamber (Coy Labs, Inc, USA) maintained at 5% carbon dioxide, 4% hydrogen, 91% nitrogen, and 37°C. Monocultures were adjusted to OD600 = 0.01 in 2 ml BHI and cultured for 0 and 24 h at 37°C without shaking, centrifuged at 3500 rpm for 10 min, the supernatant filtered at 0.22 μm and aliquots stored at −80°C until analysis via ultrahigh performance liquid chromatography-tandem mass spectroscopy (UPLC-MS/MS) (Metabolon Inc. Durham, NC). Medium only bacteria-free cultures were processed and analyzed in tandem as a control. Bacterial pellets were reserved for quantification using species-specific primers as described previously.^68,91,92^ *In vitro* cultures were conducted in technical triplicate. Endpoint qPCR and metabolomics analyses were conducted in duplicate.

### Metabolomics

Bacterial cell-free supernatants were shipped on dry ice to Metabolon Inc. Durham, NC, for UPLC-MS/MS.^93^ Briefly, proteins were precipitated with methanol by shaking for 2 min, and centrifugation followed division into five fractions, removal of the solvent with a TurboVap, and storage overnight under nitrogen. The dried extract was reconstituted by: 1) elution from a C18 column (Waters UPLC BEH C18–2.1 × 100 mm, 1.7 µm) with water and methanol, containing 0.05% perfluoropentanoic acid (PFPA) and 0.1% formic acid (FA) for acidic positive ionization of hydrophilic compounds, 2) elution from the same C18 column using methanol, acetonitrile, water, 0.05% PFPA, and 0.01% FA for acidic positive ionization of hydrophobic compounds 3) elution from a separate C18 column using methanol and water with 6.5 mm ammonium bicarbonate at pH8 for basic negative ionization and 4) elution from a HILIC column (Waters UPLC BEH Amide 2.1 × 150 mm, 1.7 µm) using a gradient consisting of water and acetonitrile with 10 mm ammonium formate, pH 10.8 for negative ionization. All methods used ultra-performance liquid chromatography (UPLC) and high resolution/accurate mass spectrometer with heated electrospray ionization and 35,000 mass resolution using an Orbitrap mass analyzer. Analysis included iterative mass spectrometry and data-dependent tandem mass spectrometry scans using dynamic exclusion and a range of 70–1000 m/z. Raw data was extracted for peak identification and quality assessment using Metabolon’s hardware and software using a proprietary library of 3300 authenticated purified standards. Resulting data was analyzed using MetaboAnalyst, version 6.0.^94^ All data was log-transformed and mean centered with missing values excluded. Statistical analysis included one-way ANOVA at a threshold of *p* ≤ 0.05 and |FoldChange|>1.5 with post-hoc analyses using Fisher's least significant difference (LSD).

### Butyric acid quantification

Gas chromatography coupled with mass spectrometry (GC-MS) was used to quantify butyrate levels in fecal samples. Fecal samples were directly weighed into a 2 mL microcentrifuge tube, and 700 mg silica beads and 600 µL distilled and deionized water were added to each. Samples were homogenized using a bead beater. After homogenization, samples were centrifuged at 10,000 rpm for 10 min and the liquified sample in the supernatant was extracted. Solvent extraction of butyric acid was performed by combining 200 µL of supernatant, 20 µL of hydrochloric acid (HCl), 100 µL of potassium bisulfate (KHSO4) and 1 mL of dimethyl carbonate (DMC) into a 2 mL microcentrifuge tube.^95^ Upon addition of HCl, butyrate is protonated to butyric acid which readily partitions into the DMC. The resulting mixture was vortexed for 10 s and centrifuged at 3800 rpm for 10 min. The supernatant organic phase was then transferred into a GC-MS vial for analysis. For the quantification of butyric acid, a Shimadzu Nexus GS 2030 couple to a TQ8040NX mass spectrometer was used. The GC-MS autosampler used a 2 μL smart syringe to inject 0.8 μL of liquid sample. A DB-FATWAX UI column, with 30 m length, 0.25 μm thickness, and 0.23 mm diameter was used. Upon injection of the sample, the oven temperature was held at 80°C for 1 min and then increased by 15°C/min until it reached 115°C. The oven temperature was held at 115°C for 3 min. Then, the oven temperature ramped again at a rate of 3°C/min until it reached 130°C. The temperature was then increased at a rate of 15°C/min until it reached 230°C. The oven was held at 230°C for 3 min. Acquisition mode was Q3 scan, with ion source temperature and interface temperature at 280°C and 250°C, respectively. Concentrations were determined using a calibration curve generated from external standards that underwent the same extraction protocol as samples. Concentrations were then used to calculate mg of butyrate per gram of fecal matter.

### Bacteriocin identification

Whole-genome sequencing and assembly of *L. reuteri* and *L. murinus* isolates have been described previously.^68^ Briefly, DNA was extracted from two pure isolates per *Lactobacillaceae* member using the UltraClean Microbial Kit (Qiagen, USA), quality assessed using Qubit and 2100 Bioanalyzer High Sensitivity DNA analysis, fragmentation-free libraries prepared using a Ligation Sequencing Kit (SQK-LSK10) and Native Barcoding Expansion (EXP-NBD104, Nanopore, USA) for multiplexing and sequencing on a Nanopore GridIon X5 Long Read Sequencer (Flow Cell ID, FAL58627, Nanopore, USA) with basecalling using Guppy version 3.2.8. Sequencing quality was assessed using NanoQC and Nanoplot version 1.0.1, filtered at a quality score of 7, trimmed using Trimmomatic version 0.39 and assembled using Flye version 2.6. Bacteriocins were predicted from draft genomes using BAGEL4.^96^ Draft genomes are publicly available as described previously.^68^

## Results

### Lactobacillaceae *differentially impact CNS autoimmunity*

**Figure 1.**
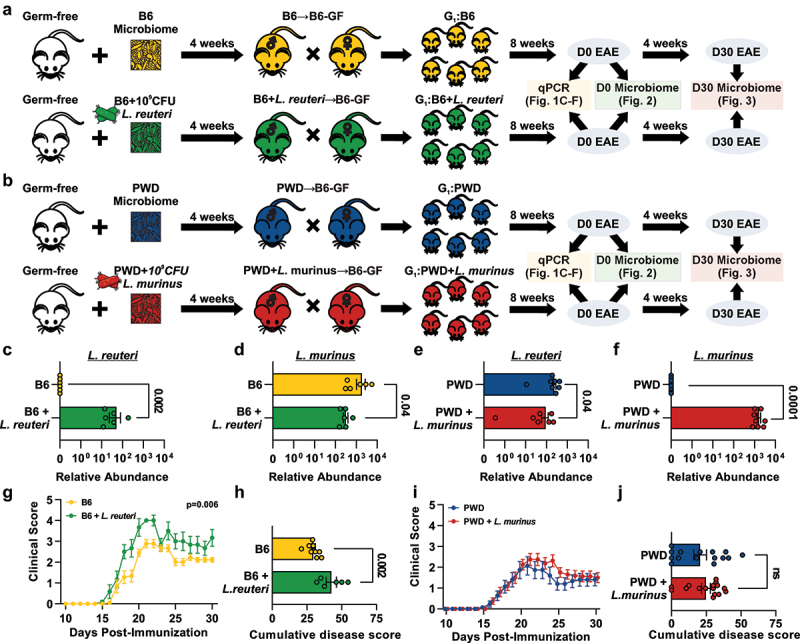
*Lactobacillaceae* family members differentially impact CNS autoimmunity. Diagram of experimental microbiome manipulation and breeding schemes wherein ex-germ-free mice received (a) the B6 gut microbiome naturally devoid of *L. reuteri* or supplemented with 10^9^ CFU *L. reuteri* or (b) the PWD gut microbiome naturally lacking *L. murinus* or PWD + 10^9^ CFU *L. murinus*. Experimental microbiomes were introduced as a one-time inoculation in ex-gf breeding pairs for vertical transmission of the gut microbiota to experimental offspring. Relative abundance of *L. reuteri* and *L. murinus* in each gut microbiome configuration as determined by species-specific qPCR is shown in (c–f). EAE was induced in experimental offspring at 8–12 weeks of age in mice colonized with the B6 (*n* = 9) and B6 + *L. reuteri* (*n* = 6) gut microbiome (g) or the PWD (*n* = 15) and PWD + *L. murinus* (*n* = 16) gut microbiome (i). Cumulative disease score (CDS) representing the total sum of all daily EAE scores over a 30-day disease course for each gut microbiome configuration is shown in (h) and (j) with significance evaluated using Mann–Whitney nonparametric testing.

In our previous studies, we found that transplantation of gut microbiota from wild-derived PWD mice into GF B6 mice resulted in exacerbated EAE severity compared with microbiota derived from B6 mice. We determined that this was in part mediated by the transfer of *L. reuteri* native to the PWD microbiota. We also observed that the PWD microbiome was colonized with a high abundance of *Limosilactobacillus reuteri* (*L. reuteri*) with an absence if *Ligilactobacillus murinus* (*L. murinus*), whereas in B6 microbiota the inverse relationship was observed.^97^ To interrogate the interplay between divergent gut microbiome compositions and the presence of specific *Lactobacillaceae*, we isolated and cryopreserved both *Lactobacillaceae* members of interest and their originating gut microbiomes (B6 and PWD), and colonized GF mice to establish breeding pairs with four distinct sets of gut microbiota configurations: 1 & 2) the B6 gut microbiome (naturally devoid of *L. reuteri*), co-colonized or not with 10^9^ CFU *L. reuteri*, and 3 & 4) the PWD gut microbiome (naturally lacking *L. murinus*), co-colonized or not with 10^9^ CFU *L. murinus*, to thus complement the *Lactobacillaceae* members that were naturally absent (Figure 1a,b). Stable colonization of *Lactobacillaceae* members and subsequent vertical transmission to offspring was confirmed using qPCR with species specific primers (Figure 1c-f). Introduction of either *L. reuteri* or *L. murinus* into the B6 or PWD gut microbiome, respectively, resulted in a significant decrease (*p* = 0.04) (Figure 1d,e) of the other *Lactobacillaceae* member, indicating potential niche competition between these closely related species. To determine the impact of *Lactobacillaceae* colonization on CNS autoimmunity, we induced EAE in resulting vertically colonized offspring. Introduction of *L. reuteri* into the B6 microbiome significantly exacerbated EAE, as we have shown previously,^68,69^ as indicated by disease course over time (*p* = 0.006) and cumulative disease score (CDS) (*p* = 0.002) (Figure 1g,h). In contrast, the introduction of *L. murinus* into the PWD microbiome did not significantly impact EAE severity (Figure 1i,j). Notably, the finding that *L. murinus* colonized the PWD microbiome at a higher level than that observed with *L. reuteri* colonization in the B6 gut microbiome (*p* = 0.006) (Figure 1c,f), suggests that the differential impacts on EAE severity are not driven by the differences in abundance or colonization efficiency. Taken together, these results suggest that distinct members of *Lactobacillaceae* have differential impacts on CNS autoimmunity.

### Lactobacillaceae *elicit specific alterations in gut microbiota composition*

**Figure 2.**
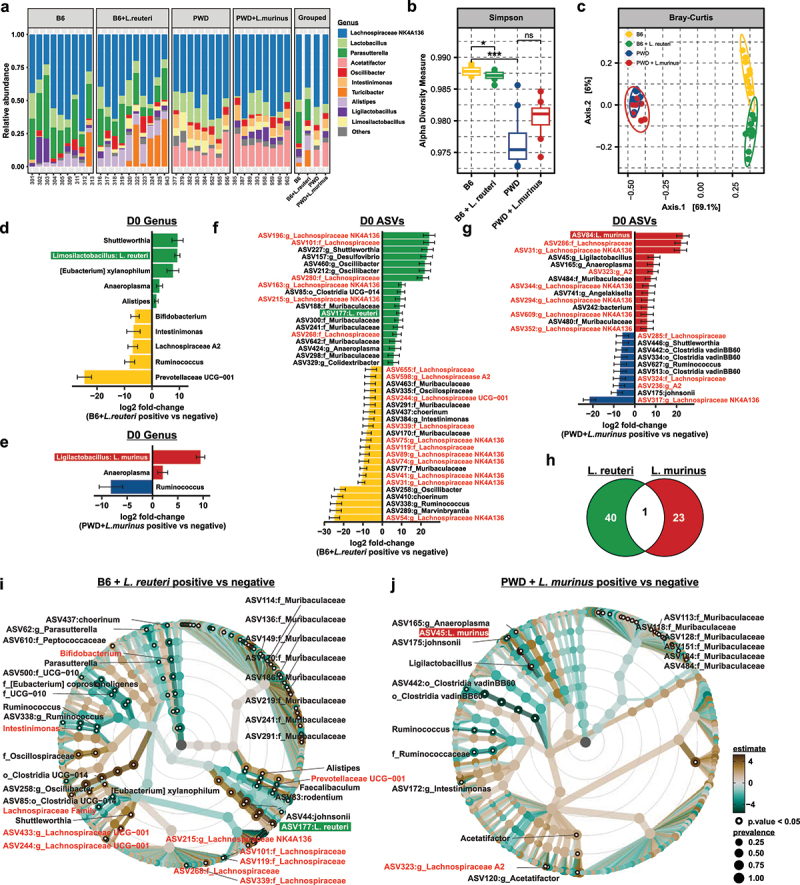
*Lactobacillaceae* differentially impact gut microbiome composition. (a) Stacked bar plots of top 11 most abundant genera as a proportion of total 16S V3V4 amplicon reads. (b) Alpha (Simpson) and (c) beta (Bray–Curtis dissimilarity) diversity analysis in each gut microbiome configuration in baseline (D0) fecal samples with significance analyzed using Wilcoxon rank sum non-parametric or Adonis testing, respectively. Differentially abundant genera and ASVs represented as a taxonomic best-hit between the B6 (*n* = 9) and B6 +* L. reuteri* (*n* = 10) (d and f) or PWD (*n* = 8) and PWD + *L. murinus* (*n* = 8) (e and g) gut microbiomes at the D0 timepoint as determined by DESeq2 using a cutoff of p_adj_ ≤0.05. Log2 fold-change reflects increased abundance in the presence of each respective *Lactobacillaceae* member when positive, and decreased abundance when negative. (h) Venn diagrams comparing differentially abundant ASVs as shown in (f) and (g) between the B6 +* L. reuteri* and PWD + *L. murinus* gut microbiome contexts. Taxonomic association heat trees comparing differentially abundant microbiota between the B6 and B6 + *L. reuteri* (i) or PWD and PWD + *L. murinus* (j) microbiomes as determined by total sum scaling (TSS) log2 linear regression. Data was filtered at a minimum prevalence of 0.05, significance determined at a cutoff of p_adj_ ≤0.05. Node size indicates proportional prevalence where significant nodes are represented as open circles, warmer colors are enriched in the presence of each *Lactobacillaceae* member, with cooler colors indicating depletion.

Because *Lactobacillaceae* are known to alter the abundance of other members of the gut microbiota,^98–100^ we posited that the disparate effects of *L. reuteri* and *L. murinus* on CNS autoimmunity might result from divergent impact on the overall gut microbiota community structure. To determine the extent to which *Lactobacillaceae* members differentially impact the composition of the gut microbiome, we collected fecal samples for gut microbiome profiling via 16S sequencing at two distinct timepoints: 1) prior to EAE induction (D0) and 2) following a full 30-day disease course (D30). We initially restricted our analyses to the D0 timepoint to establish the impact of each *Lactobacillaceae* member on the baseline gut microbiota, to highlight a potential role in disease initiation, rather than progression. To explore major changes in gut microbiota community structure in the presence or absence of *L. reuteri* or *L. murinus*, we visualized of the top 11 most abundant genera as a bar graph (Figure 2a). Consistent with our previous findings, the B6 and PWD gut microbiota were highly divergent even after transplantation into B6 recipients, with the B6 microbiome containing *Parasutterella*, *Turicibacter*, and *Alistipes*, while the PWD microbiome was colonized by *Acetatifactor* and *Intestinimonas*. To determine the extent to which each *Lactobacillaceae* member impacts gut microbiome composition, we next analyzed metrics of intra- and inter-sample gut microbial diversity. Simpson’s alpha diversity, as a metric of species richness accounting for both the number of species present and their relative abundance, recapitulated our previous findings, with the B6 microbiome displaying greater species richness as compared to the PWD microbiome (*p* < 0.001) (Figure 2b and Table S1).^97^ When introduced into the B6 gut microbiome, *L. reuteri* modestly but significantly contracted species richness (*p* < 0.05), while *L. murinus* did not (*p* = 0.11) (Figure 2b and Table S1). Analysis of Bray–Curtis dissimilarity as a metric of between group diversity, indicated a stark difference in composition of the B6 and PWD gut microbiomes (R^2^ = 0.77, *p* = 0.006) (Figure 2c and Table S2), recapitulating our previous findings in this new cohort.^97^ Importantly, while colonization by *L. reuteri* was sufficient to significantly alter the overall structure of the gut microbiome (R^2^ = 0.26, *p* = 0.006), colonization by *L. murinus* was not (R^2^ = 0.16, *p* = 0.07) (Figure 2c and Table S2).

Given the alpha and beta diversity analysis indicating that *L. reuteri* and *L. murinus* differentially impact gut microbiome composition, we next analyzed the specific compositional changes produced by each species at each taxonomic rank by comparing: 1) B6 vs. B6 + *L. reuteri* and 2) PWD vs. PWD + *L. murinus* using DESeq2 to assess differential abundance of individual taxa (Figure 2d-g). In genus level analyses, as expected, the genus representing each experimentally introduced species was differentially abundant, with an increase in *Limosilactobacillus* in the B6 + *L. reuteri* microbiome and an increase in *Ligilactobacillus* in the PWD + *L. murinus* microbiome (Figure 2d,e, Tables S3 and S4). Strikingly, while the introduction of *L. reuteri* resulted in a reduction of *Prevotellaceae*, *Ruminococcus*, *Bifidobacterium*, *Intestinimonas*, and *Lachnospiraceae A2*, *L. murinus* colonization resulted in depletion of only a single genus (*Ruminococcus*) (Figure 2d,e, Tables S3 and S4). Differential abundance analysis at the ASV level revealed a profound modulation of *Lachnospiraceae*, where *UCG-001* and *NK4A136 groups* were also significantly impacted by the presence of *L. reuteri* (Figure 2f and Table S5). A greater number of ASVs displayed altered abundance in the context of *L. reuteri* colonization as compared to *L. murinus* (41 vs. 24), with only a single shared ASV, ASV31:g__*Lachnospiraceae NK4A136*, altered in both microbiome configurations, albeit with opposing directionality (decreased in B6+*L. reuteri* and increased in PWD+*L. murinus*) (Figure 2f-h, Tables S5 and **S6**). In a parallel approach to identify differentially abundant taxa and visualize their phylogenetic relationships, total sum scaling (TSS) log2 linear regression modeling was applied and represented as a taxonomic association heat tree. Consistent with direct differential abundance analysis, a greater number of differentially abundant taxa were observed in the context of *L. reuteri* colonization as compared to *L. murinus*, 114 (66 decreased and 48 increased) versus 38 (15 decreased and 23 increased) (Figure 2i,j, Tables S7 and S8). As above, microbiota altered by *L. reuteri* colonization were comprised of known SCFA-producing taxa, including *Prevotellaceae*, *Bifidobacterium*, *Intestinimonas*, and *Lachnospiraceae*.^101–106^ These results demonstrate that colonization by *L. reuteri* impacts the gut microbial community composition to a greater extent than does colonization by *L. murinus*, with a notable impact on the abundance of SCFA-producers.

### L. reuteri *drives persistent remodeling of the gut microbiome during autoimmune disease*

**Figure 3.**
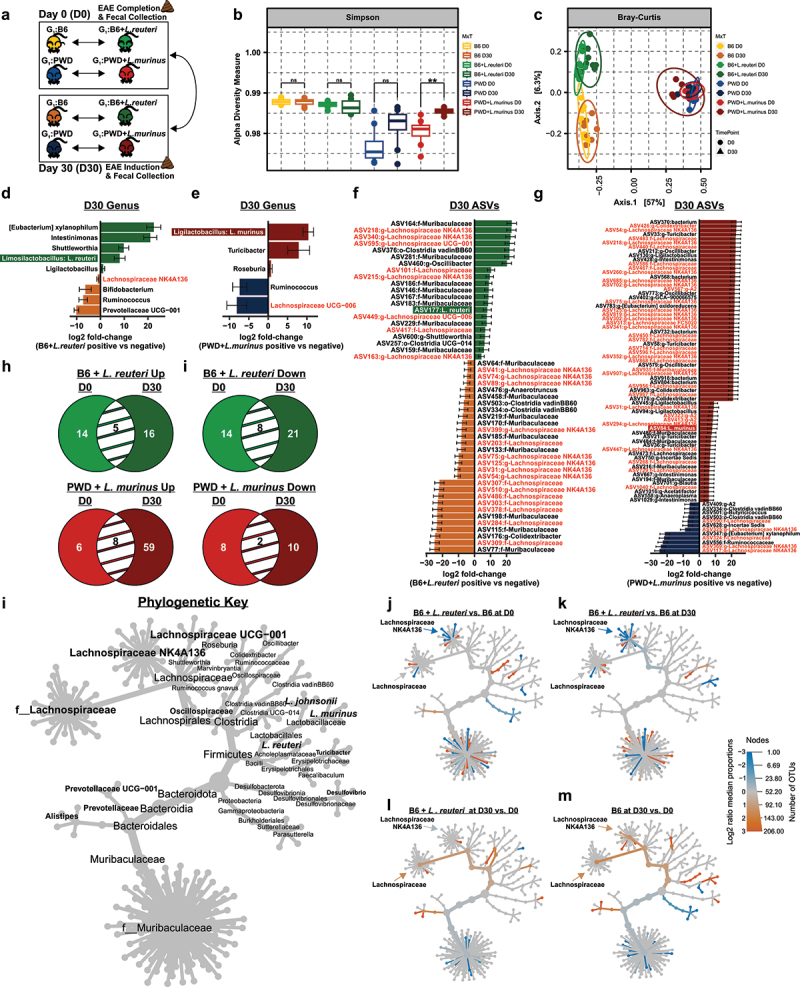
*L. reuteri* drives persistent remodeling of the gut microbiome during autoimmune disease. (a) Diagram depicting experimental gut microbiomes and fecal collection timepoints relative to EAE induction. (b) Alpha (Simpson) and (c) beta (Bray-Curtis dissimilarity) diversity analysis in each gut microbiome configuration comparing D0 and D30 fecal samples with significance analyzed using Wilcoxon rank sum non-parametric or Adonis testing, respectively. Differentially abundant genera and ASVs represented as a taxonomic best-hit between the B6 (*n* = 9) and B6 + *L. reuteri* (*n* = 10) (d and f) or PWD (*n* = 8) and PWD + *L. murinus* (*n* = 6) (e and g) gut microbiomes at the D30 timepoint as determined by DESeq2 using a cutoff of p_adj_ ≤0.05. Log2 fold-change reflects increased abundance in the presence of each respective *Lactobacillaceae* member when positive, and decreased abundance when negative. Venn diagrams comparing enriched (h) or depleted (i) ASVs between the D0 and D30 timepoints in the B6 + *L. reuteri* and PWD + *L. murinus* gut microbiomes. Heat trees comparing differentially abundant taxa between gut microbiome contexts (j) and (k) and over time (l) and (m) with phylogenetic key show in (i) determined by Wilcoxon rank sum non-parametric testing of the median abundance of taxa. Node color represents the log2 media proportion of differentially abundant genera at padj < 0.05.

To determine if the observed *Lactobacillaceae*-driven changes in gut microbiome composition were preserved over time and in the context of ongoing CNS autoimmunity, we next analyzed the composition of the gut microbiome following a full 30-day disease course (D30) in the EAE model by comparing the B6 vs. B6 + *L. reuteri* and PWD vs. PWD + *L. murinus* gut microbiomes at this timepoint and contrasting these results to the D0 timepoint (Figure 3a). Alpha diversity analysis demonstrated no significant difference in intra-individual gut microbial diversity between the D0 and D30 timepoint in the context of *L. reuteri* colonization, with a subtle but significant expansion of diversity between D0 and D30 with *L. murinus* colonization (Figure 3b and Table S9). Beta diversity analysis at the D30 timepoint, as measured by Bray-Curtis dissimilarity, indicated conservation of not only compositional differences between the B6 and PWD gut microbiomes, but also compositional differences driven by *L. reuteri* colonization (Permanova, effect of *L. reuteri* R^2^ = 0.63, *p* < 0.001) with more subtle shifts over time (Permanova, effect of time R^2^ = 0.02, *p* = 0.02) in a baseline microbiome-dependent fashion (Permanova, effect of L. reuteri*time interaction R^2^ = 0.03, *p* = 0.03) (Figure 3c and Table S10). In contrast, *L. murinus* colonization did not significantly affect overall composition at the day 30 timepoint (Permanova, effect of *L. murinus* R^2^ = 0.20, *p* = 0.22) or over time between the D0 and D30 timepoints (Permanova, effect of time R^2^ = 0.22, *p* = 0.06) (Table S10). These data suggest the *L. reuteri* driven shifts in gut microbial community structure observed prior to EAE induction (D0), were preserved during ongoing EAE (D30).

To interrogate the specific nature of gut microbial shifts occurring by D30 post-EAE, as potential drivers of disease severity (rather than disease initiation) or occurring as a consequence of disease state, we next analyzed the specific compositional changes produced by colonization with each *Lactobacillaceae* species at each taxonomic rank by comparing: 1) B6 vs. B6 + *L. reuteri* and 2) PWD vs. PWD + *L. murinus*, at the D30 timepoint, using DESeq.^83^ At the taxonomic rank of genus, consistent with D0 analyses, a reduction of potential SCFA-producing microbiota, including *Lachnospiraceae NK4A136*, *Bifidobacterium*, and *Prevotellaceae*, was observed in the B6 + *L. reuteri* gut microbiome (Figure 3d and Table S11). Interestingly, a marked depletion of a distinct clade of *Lachnospiraceae UCG-006*, was also observed by D30 in the context of the PWD + *L. murinus* microbiome (Figure 3e and Table S12). ASV level analyses revealed a marked depletion of *Lachnospiraceae* ASVs from several distinct clades in the context of *L. reuteri* colonization (Figure 3f and Table S13). By contrast, many *Lachnospiraceae* ASVs were increased, rather than decreased, by *L. murinus* colonization (Figure 3g and Table S14). A direct comparison of differentially abundant ASVs driven by either *Lactobacillaceae* member at D0 and D30 revealed partial overlap, with a large expansion of individual ASVs by D30 with *L. murinus* colonization (67 versus 21 ASVs) (Figure 3H, I, Tables S13 and S14), consistent with increased alpha diversity in this gut microbiome configuration by D30 (Figure 3b).

Differential abundance analyses above suggested that clade-specific alteration of *Lachnospiraceae* may result from colonization by individual *Lactobacillaceae* members. To assess this possibility, we used a parallel method to identify statistically disparate taxa by Wilcoxon Rank Sum test of median abundance of each taxonomic rank at p_adj_ ≤0.05 by pairwise comparisons. Resulting data was visualized as differential abundance heat trees accounting for phylogenetic relationships, microbiome configuration, and timepoint. Comparing the B6 and B6 + *L. reuteri* microbiomes over time revealed that while the *Lachnospiraceae NK4A136* clade was depleted in the context of *L. reuteri* colonization at D0, the *Lachnospiraceae* clade was not (Figure 3j and Table S15), and that this effect was more pronounced by D30 (Figure 3k and Table S15). Importantly, over time, while there was a pronounced increase within both *Lachnospiraceae* clades in the B6 microbiome by D30 (Figure 3m and Table S15), *NK4A136* was not enriched following EAE in the B6 + *L. reuteri* gut microbiome at this timepoint (Figure 3l and Table S15). By contrast, a limited number of taxa were altered between the PWD and PWD + *L. murinus* gut microbiome states, with the exception of a noted expansion of both *Lachnospiraceae* and *Lachnospiraceae NK4A136* by D30 (Figure S1 and Table S16), as consistent with differential abundance analysis (Figure 3g). These data suggest that *L. reuteri* drives a sustained clade-specific contraction of *Lachnospiraceae* over time, while *L. murinus* exerts a more subtle and opposite effect.

### Lactobacillaceae *differentially impact the functional potential of the gut microbiota*

**Figure 4.**
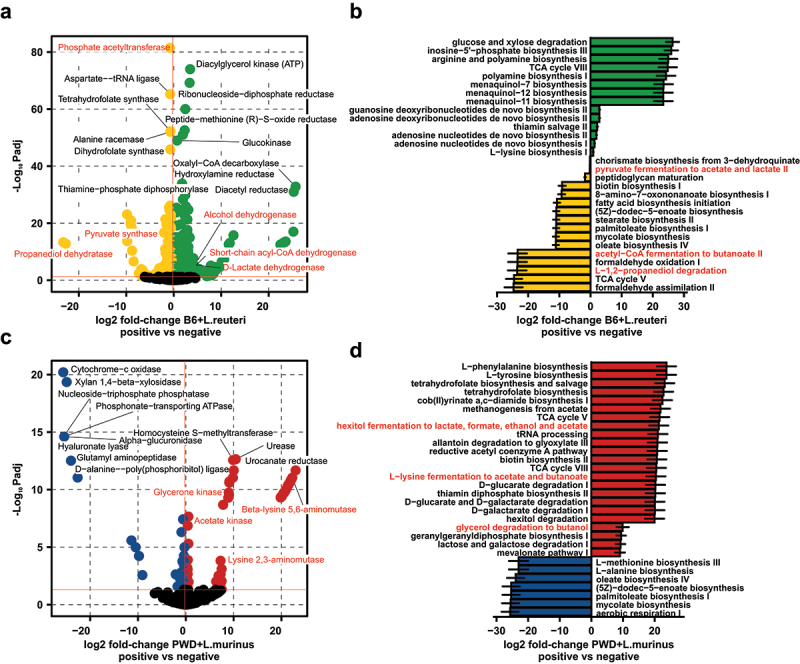
*Lactobacillaceae* differentially impact the functional potential of the gut microbiota. Volcano plot of differentially abundant enzymes (a and c) and fold-change of pathways (b and d) in the inferred metagenome of the B6 + *L. reuteri* (a and b) and PWD + *L. murinus* (c and d) gut microbiomes at the day 30 timepoint. Metagenomic content was inferred using PICRUSt2 with differential abundance analyzed for the subset of enriched and depleted ASVs using DESeq2 at p_adj_ ≤0.05.

To begin to assess the functional consequences of *Lactobacillaceae-*mediated shifts in gut microbiome composition, we performed pathway enrichment analysis using *PICRUSt2*^88,89^ to infer the metagenomic potential of those ASVs found to be over- or under-represented upon colonization with *L. reuteri* or *L. murinus*. ASVs modulated by *L. reuteri* colonization were predicted to encode a number of enzymes involved in SCFA production (Figure 4a and Table S17), with pathway enrichment analysis indicating a global depletion of acetate, propionate, and butyrate (a.k.a. butanoate) metabolism (Figure 4b and Table S18). By contrast, those ASVs found to be enriched or depleted by *L. murinus* colonization displayed the opposite inferred metagenomic potential, with both enzymes and pathways involved in acetate and butyrate displaying enrichment (Figure 4c,d, Tables S19 and S20).

**Figure 5.**
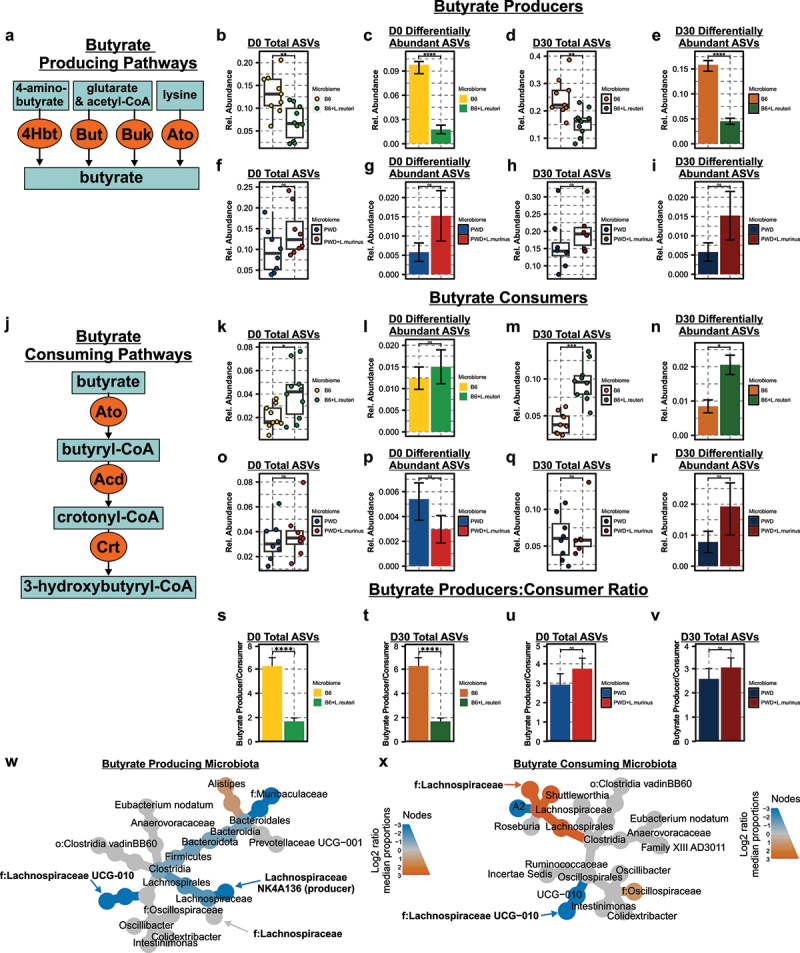
*L. reuteri* alters the abundance of butyrate-producing and -consuming microbiota. (a) Schematic of microbial butyrate-producing pathways and terminal enzymes. Relative abundance of butyrate-producing microbiota in the B6 and B6 +* L. reuteri* total gut microbiome at D0 (b) and D30 (d) and in differentially abundant microbiota at the D0 (c) and D30 (e) with significance analyzed using Wilcoxon rank sum non-parametric testing. Relative abundance of butyrate-producing microbiota in the PWD and PWD + *L. murinus* total gut microbiome at D0 (f) and D30 (h) and in differentially abundant microbiota at the D0 (g) and D30 (i) with significance analyzed using Wilcoxon rank sum non-parametric testing. (j) Schematic of the microbial butyrate consuming pathway. Relative abundance of butyrate consuming microbiota in the B6 and B6 +* L. reuteri* total gut microbiome at D0 (k) and D30 (m) and in differentially abundant microbiota at the D0 (l) and D30 (n) with significance analyzed using Wilcoxon rank sum non-parametric testing. Relative abundance of butyrate consuming microbiota in the PWD and PWD + *L. murinus* total gut microbiome at D0 (o) and D30 (q) and in differentially abundant microbiota at the D0 (p) and D30 (r) with significance analyzed using Wilcoxon rank sum non-parametric testing. The ratio of butyrate producers to consumers in the B6 and B6 + *L. reuteri* gut microbiomes at D0 (s) and D30 (t) and PWD and PWD + *L. murinus* at D0 (u) and D30 (v) gut microbiomes with significance analyzed using Wilcoxon rank sum non-parametric testing. Heat trees of differentially abundant butyrate producing (w) and consuming (x) microbiota between the B6 and B6 +* L. reuteri* gut microbiomes with significance determined by Wilcoxon rank sum non-parametric testing of the median abundance of taxa. Node color represents the log2 media proportion of differentially abundant genera at padj < 0.05.

Given the mounting evidence for the importance of butyrate in MS and other autoimmune diseases,^16–18,107^ the observed differential abundance of potential SCFA-producing microbiota (Figures 2 and 3), and divergent predicted effects on butyrate metabolism (Figure 4), we next sought to formally define butyrate-producing and consuming enzymes, pathways, and associated microbiota which were altered in response to *L. reuteri* colonization. Previous studies have defined four independent pathways associated with terminal butyrate-producing enzymes conserved among distinct clades of microbiota, stemming from 4-amino-butyrate, glutarate, and acetyl-CoA, or lysine metabolism (Figure 5a).^60^ Terminal enzymes responsible for final butyrate production within each pathway were quantified as described in Methods, to compare global representation of this metabolic function. The relative abundance of total butyrate-producing microbiota (i.e., microbiota encoding enzymes for butyrate production) was decreased in by *L. reuteri* colonization at D0 (Figure 5b). Subsetting this analysis to the ASVs identified as differentially abundant in the presence of *L. reuteri* also demonstrated a significant depletion in butyrate-producing microbiota (Figure 5c). Depletion of butyrate-producing microbiota in the context of *L. reuteri* colonization was sustained at the D30 timepoint (Figure 5d,e). By contrast, *L. murinus* colonization elicited no significant change in total or differentially abundant butyrate producers at either timepoint (Figure 5f-i).

Butyrate-consuming pathways require a series of three essential enzymes, including butyryl-CoA:acetoacetate CoA transferase (Ato), acyl-CoA dehydrogenase/butyryl-CoA dehydrogenase (Acd), and enoyl-CoA hydratase/crotonyl-CoA hydratase (Crt) (Figure 5j).^61^ The relative abundance of microbiota (as a proportion of total microbiota) that encoded these required enzymes (i.e., butyrate consumers) was elevated in the presence of *L. reuteri* (*p* ≤ 0.05) (Figure 5k), although this was not significant when subsetted to differentially abundant microbiota at D0 (Figure 5l). However, by the D30 timepoint, *L. reuteri* colonization led to a significant enrichment in butyrate consumers in both the relative abundance of total and differentially abundant microbiota (Figure 5m,n). *L. murinus* did not significantly alter the relative abundance of butyrate consumers at either timepoint (Figure 5o-r). As a metric of net butyrate-producing potential of a gut microbiome configuration at a given timepoint, we next calculated the ratio of butyrate producer to consumer abundance within total ASVs. Colonization by *L. reuteri* caused a marked reduction in the producer:consumer ratio at both D0 and D30 (Figure 5s,t). Notably, *L. murinus* colonization did not exert the same effect, yielding no significant difference in producer:consumer ratio at D0 and D30 (Figure 5u,v). Taken together, these results suggest that colonization by *L. reuteri* strongly depletes butyrate-producing microbiota and modestly expands butyrate-consuming microbiota, while *L. murinus* does not.

To identify specific members of, and phylogenetic relationships between, the gut microbiota responsible for potential differential butyrate production or consumption that are altered by *L. reuteri* colonization, taxa predicted to encode each butyrate-associated enzyme (Figure 5a,j) were extracted and represented as a phylogenetic heat tree. Among butyrate producers, *Lachnospiraceae NK4A136* were depleted in the presence of *L. reuteri* (Figure 5w and Table S22), while an enrichment of a distinct sub-clade of *Lachnospiraceae* was observed among butyrate consumers (Figure 5x and Table S23). Interestingly, depletion of *Lachnospiraceae UCG-010* occurred within both butyrate producers and consumers (Figure 5w,x). These data suggest that *L. reuteri* colonization modulates multiple distinct clades of SCFA-producing and consuming *Lachnospiraceae*, with a predicted net effect of total butyrate depletion.

### L. reuteri *depletes SCFA and their producers* in vitro

**Figure 6.**
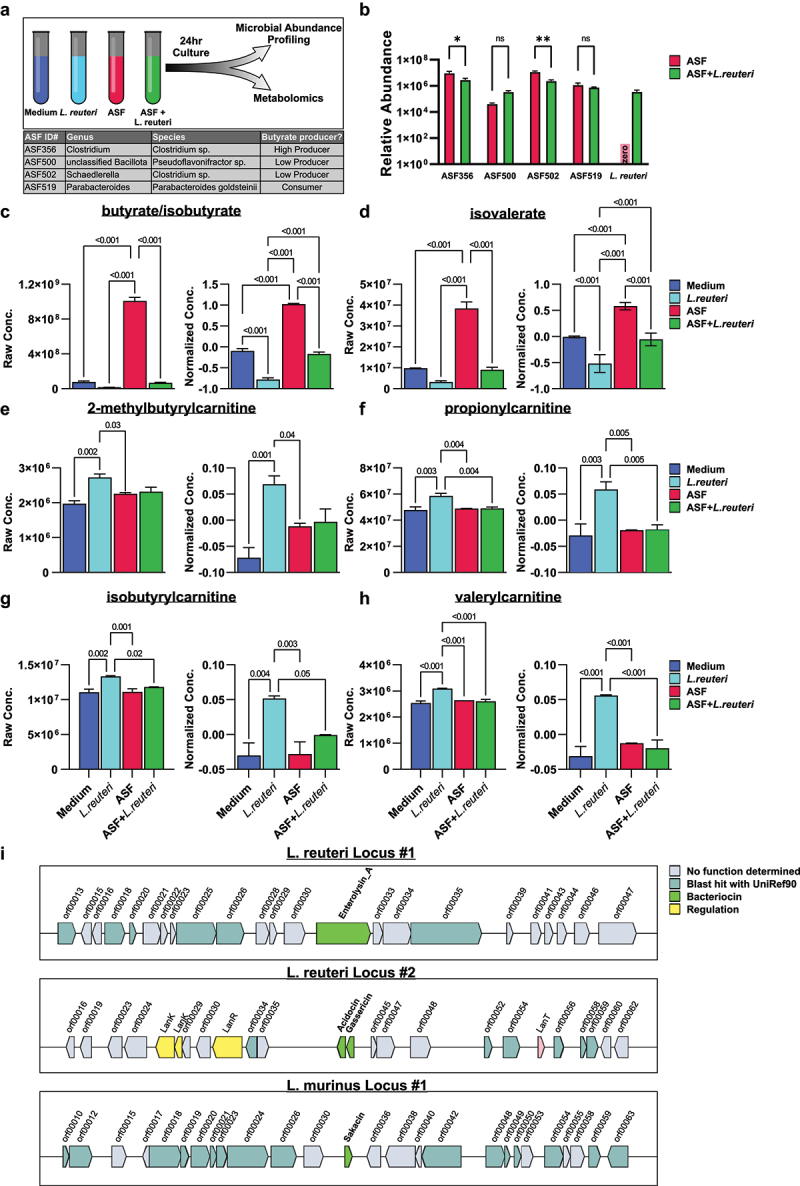
*L. reuteri* modulates SCFA abundance through direct and indirect mechanisms. (a) Schematic depicting in vitro co-cultures of the ASF4 minimal microbiome and *L. reuteri*. (b) Relative abundance of the ASF at 24 hrs when cultured with and without *L. reuteri* as determined by species-specific qPCR. (c-h) SCFAs and associated metabolites in cell free supernatants analyzed using one-way ANOVA with post-hoc testing using Fisher’s LSD and represented as log-transformed and mean centered abundance profiles. (i) Loci predicted to encode putative bacteriocins in the genomes of *L. reuteri* and *L. murinus* isolates. In vitro cultures were conducted in technical triplicate. Endpoint qPCR and metabolomics analyses were conducted in duplicate.

Accurate measurement of SCFAs *in vivo* is challenging due to their rapid absorption and metabolism in a mammalian host.^108^ Therefore, to determine if *L. reuteri* colonization is sufficient to alter the abundance of SCFA-producing microbiota and/or their production of SCFAs, we leveraged *in vitro* culture of a simplified 4-species version of the Altered Schaedler’s flora (ASF4) as a model minimal defined microbiome, which included several species that are known to produce SCFAs, including two members of the *Lachnospiraceae* family, ASF356 and ASF502 (Figure 6a). Cultures of *L. reuteri* alone, ASF4, or ASF4 + *L. reuteri* were grown anaerobically in brain heart infusion medium for 24 h, followed by quantification of individual bacterial species abundance by qPCR and metabolic profiling via ultrahigh performance liquid chromatography-tandem mass spectroscopy (UPLC-MS/MS) (Table S24). Following 24 h of culture, the abundance of two species of the ASF known to produce butyrate, ASF356, a member of *Clostridiaceae*, and ASF502, a member of *Lachnospiraceae*,^109,110^ were reduced in the presence of *L. reuteri*, while the levels of ASF500, a low butyrate producer, and ASF519 which is both butyrate producers and consumer were not significantly affected (Figure 6b). Consistent with this, the minimal ASF4 consortium produced significant levels of butyrate, with marked depletion when grown together with *L. reuteri* (Figure 6c). Interestingly, when cultured alone, *L. reuteri* also appeared to deplete butyrate present in the basal culture medium, indicating active consumption of butyrate by this species (Figure 6c). While acetate and propionate were below the size threshold for detection in our mass spectrometry analysis, isovalerate, a 5-carbon SCFA, was also produced by the ASF and markedly depleted by *L. reuteri* in monoculture (Figure 6d). Although bacteria do not directly synthesize carnitine, they readily produce acyl-carnitines (including, carnitine conjugated SCFAs) as carbon, nitrogen, and energy sources.^111^ Consistent with the notion of acyl-carnitine conjugation as a nutrient sequestration method for *L. reuteri*, carnitine-conjugated 2-methyl-butyrate, propionate, isobutyrate, and valerate were elevated in *L. reuteri* monoculture compared to basal medium alone (Figure 6e-h). Together, these results demonstrate that *L. reuteri* can inhibit the growth of SCFA-producing microbiota and profoundly diminish net production of butyrate and isovalerate, by depletion of their microbial producers and/or direct sequestration/utilization.

To determine how *L. reuteri* might lead to active depletion of SCFA-producing microbiota, we leveraged our previously described whole-genome sequencing and assembly of two independent isolates of *L. reuteri* and *L. murinus* used in our colonization and *in vitro* culture studies.^68^ We mined draft genomes of each isolate for loci encoding bacteriocins, small peptides with broad antimicrobial activity, using BAGEL4.^96^ Genomes of *L. reuteri* isolates encoded a total of three genes predicted to encode bacteriocins, including enterolysin A, acidocin, and gassericin, while *L. murinus* contained only one, sakacin (Figure 6i). Importantly, both gassericins and acidocins are active against *Clostridial* members.^112–115^ Interestingly, neither enterolysin A, encoded by *L. reuteri*, nor the singular bacteriocin predicted to be encoded by *L. murinus*, sakacin, have been previously shown to be active against *Clostridia* or other SCFA-producing microbial species.^116–118^ These results identify a potential antimicrobial pathway encoded by *L. reuteri* which could drive competition with other gut bacteria, including SCFA-producers, to be functionally validated in future studies.

### L. reuteri-*driven exacerbation of CNS autoimmunity is dependent on dietary fiber bioavailability*

**Figure 7.**
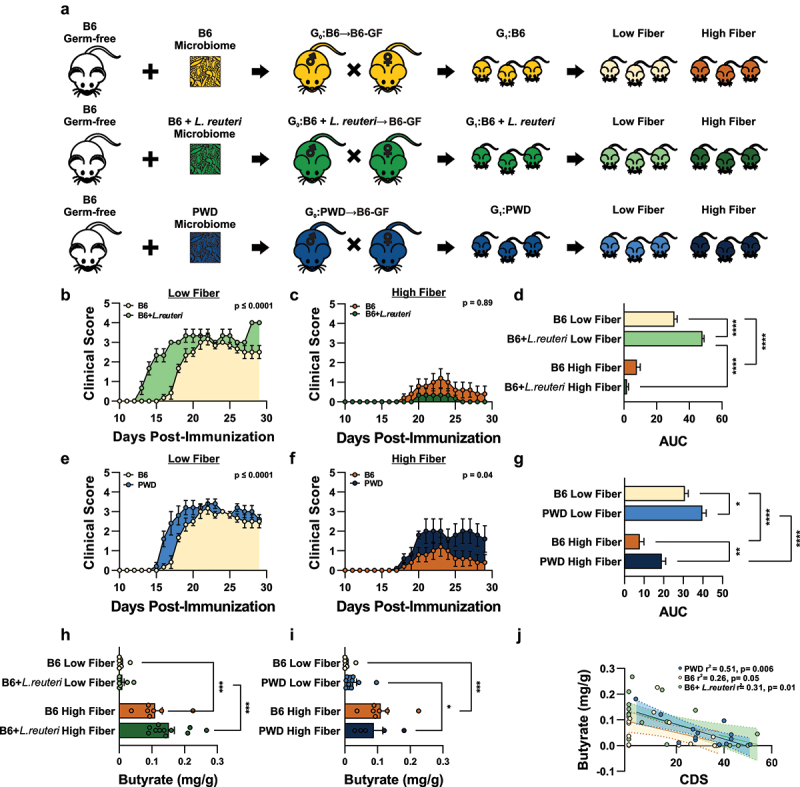
Dietary fiber and gut microbiome context modulate *L. reuteri*-driven exacerbation of CNS autoimmunity. (a) Schematic of gut microbiome transplantation model and dietary fiber intervention strategy wherein experimental offspring colonized with the B6 (*n* = 15) (naturally devoid of *L. reuteri*), B6 + *L. reuteri* (*n* = 20) (supplemented with exogenous *L. reuteri*), or PWD (*n* = 13) (where *L. reuteri* is naturally present) gut microbiomes were randomized to a low (TD00102, 0% fermentable fiber) or high (TD200242, 20% inulin, 10% pectin) fiber diet two weeks prior to EAE induction at 8-12 weeks. (b–g) EAE disease courses during dietary intervention in each gut microbiome context represented as mean daily clinical score with overall significance determined by Friedman’s non-parametric two-way ANOVA and one-way ANOVA of area under the curve (AUC). (h-i) butyrate levels in D30 fecal samples measured via GC-MS and analyzed using one-way ANOVA. (j) Association of fecal butyrate levels with CDS within each experimental microbiome context, as determined by Pearson correlation at ≥|0.2|, at padj ≤ 0.05.

To determine if depletion of SCFAs (directly or indirectly) by *L. reuteri* was responsible for the observed exacerbation of CNS autoimmunity (Figure 1c), we turned back to our established gut microbiome model (Figure 1a,b), coupled with modulation of bioavailability of dietary fiber, as the substrate necessary for microbial SCFA production (Figure 7a). Specifically, offspring from mice colonized with either the B6 (naturally devoid of *L. reuteri*), B6 + *L. reuteri* (*L. reuteri* introduced), or PWD (where *L. reuteri* is naturally occurring) microbiota were randomized to either a low (TD00102, 0% fermentable fiber) or high (TD200242, 20% inulin, 10% pectin) fiber diet 2 weeks prior to EAE induction, and maintained on experimental diets for the duration of the 30-day disease course. In mice maintained on a low fiber diet, where the substrate for SCFA production is limited, colonization of *L. reuteri* in the B6 microbiome was sufficient to exacerbate disease pathogenesis (Figure 7b,d). A high fiber diet suppressed disease in both the B6 and B6 + *L. reuteri* colonized mice, and, importantly, abrogated the ability of *L. reuteri* to exacerbate EAE (Figure 7c,d), suggesting that the effect of *L. reuteri* on EAE pathogenesis can be mitigated with the provision of excess pre-biotic substrate to SCFA-producing gut microbiota. By contrast, while colonization by the divergent PWD microbiome, containing *L. reuteri*, was sufficient to exacerbate EAE during dietary fiber restriction (Figure 7e,g), a diet high in fiber did not abrogate the effect of the PWD microbiota on promoting disease severity (Figure 7f,g). This suggests that: 1) the impact of the PWD microbiota on EAE severity may be mediated by other members of the gut microbiota whose effects are insensitive to excess SCFA, and 2) that suppression of EAE by high fiber is not absolute.

To determine if experimental diets were sufficient to modulate SCFA levels *in vivo*, butyrate levels were quantified in D30 fecal samples via gas chromatography-mass spectrometry (GC-MS). Provision of high dietary fiber, compared with low fiber, significantly increased levels of fecal butyrate in mice colonized by the B6, B6 + *L. reuteri*, and PWD microbiomes (Figure 7h,i). To determine the relationship between SCFA levels and EAE severity across different microbiome contexts and diets, we correlated levels of fecal butyrate with CDS. Levels of butyrate were most strongly correlated with CDS in the context of *L. reuteri* colonization, in mice colonized with the PWD (r^2^ = 0.51, *p* = 0.006) and B6 + *L. reuteri* (r^2^ = 0.31, *p* = 0.01) microbiomes, with a significant but less robust association in the context of the B6 microbiome where *L. reuteri* is absent (r^2^ = 0.26, *p* = 0.05) (Figure 7j). Collectively, these data suggest that *L. reuteri*-mediated EAE exacerbation is accentuated under conditions of limited dietary fiber bioavailability, consistent with *L. reuteri’s* ability to deplete SCFAs and SCFA-producing bacteria, to levels below what may be a critical threshold. In contrast, provision of excess dietary fiber is sufficient to reduce disease severity in a microbiome context-dependent manner, abrogating EAE-promoting effects by some, but not all commensal microbes.

## Discussion

Multiple case–control observational studies have reported alterations in the gut microbiome of pwMS.^44,45,49,50^ Understanding the underlying drivers of such compositional differences is challenging – without longitudinal studies to assess the gut microbiome early in the disease process, or prospective studies to evaluate the gut microbiota in populations at high risk for developing MS. Notably, such approaches are complex and can be resource intensive. Mouse models of MS, therefore, represent an attractive alternative to interrogate the drivers of altered gut microbiome composition at baseline and to evaluate their impact on disease initiation, progression, and/or severity. Here, we focused on the divergent effects of two closely related commensal *Lactobacillaceae* members, to demonstrate a differential impact on baseline gut microbiome composition that is broadly sustained through the course of EAE. Functionally, we identified a putative mechanism whereby colonization by *L. reuteri* leads to a diminished abundance of SCFA-producing microbiota and associated metabolites, in conjunction with EAE exacerbation that is accentuated under conditions of low bioavailability of host dietary fiber. By contrast, colonization by *L. murinus* did not exacerbate CNS autoimmunity and exerted an opposing effect on the gut microbiota, supporting an expansion of clades of microbiota that produce SCFAs. These data highlight a new mechanism (namely competitive interactions among the microbiota) that may account for such differences in pwMS, where SCFA and their producers are depleted.

Our data demonstrate a robust suppressive effect of high dietary fiber, as the prebiotic substrate of SCFAs sufficient to boost levels of fecal butyrate, on CNS autoimmunity. Notably, one of the most consistent metabolic signatures observed in pwMS is a reduction of SCFAs, including diminished levels of acetate, propionate, butyrate, isobutyrate, valerate, and isovalerate in feces, serum, and plasma.^16,17,54^ Early attempts at SCFA supplementation in pwMS have yielded promising results.^14^ While outcomes are still pending, a current phase 2 clinical trial evaluating the safety and efficacy of daily fiber supplements in pwMS, will couple analysis of the gut microbiota, markers of intestinal inflammation, and immunophenotyping of the blood, with relapse and remission.^119^ Importantly, this precise fiber supplement formulation being evaluated as a therapeutic in MS, has already been shown to increase beneficial SCFA-producing gut microbiota in a clinical trial treating acute COVID-19 patients.^119,120^ Known SCFA-producing microbiota including *Butyricimonas, Bacteroides, Lachnospiraceae*, and *Eubacterium*, are all reduced in the MS gut microbiome.^14–16,52,121^ Notably, our study is the first to provide a link between baseline composition of the gut microbiota and response to dietary fiber in CNS autoimmunity. In the context of excess fiber provision, while a near complete abrogation of disease pathogenesis was observed in both the B6 and B6 + *L. reuteri* microbiomes, moderate disease severity was still observed in the highly divergent PWD microbiome (Figure 7b-g), suggesting that: 1) suppression of EAE by high fiber is not absolute, and 2) that modulation of EAE by gut microbiota can occur independent of this major metabolic pathway. Interestingly, exacerbation of disease driven by *L. reuteri* was only observed in the context of low dietary fiber (Figure 7b-d), suggesting that depletion of SCFA-producing microbes by this commensal has a more profound impact on EAE under conditions where the substrate for SCFA production is limited. In concert, these data suggest the composition of the gut microbiota alters the response of CNS autoimmunity to modulation of dietary fiber and that a singular species, here *L. reuteri*, can serve as a modifier. Hence, these data imply that the composition of the microbiome may be predictive of response to therapeutic SCFAs or pre-biotic fiber, suggesting potential application as a biomarker for dietary intervention response. Moreover, these data underscore the divergent impact of closely related taxa on the baseline gut microbiome composition, its stability in the context of CNS autoimmunity, and functional consequences to the host.

Our data also highlight clade-specific nuances among putative SCFA-producers and consumers present in the gut microbiome. While global depletion of SCFA-producers is observed in response to introduction of *L. reuteri* into the gut microbiome, including depletion of *Lachnospiraceae*, *Prevotellaceae*, and *Bifidobacterium* (Figure 2d,f,i), functional evaluation of the respective contributions of individual taxonomic units to SCFA production revealed contraction of *Lachnospiraceae NK4A136*, identified as SCFA-producers, with an expansion of a distinct *Lachnospiraceae* clade, identified to be SCFA-consumers (Figure 5i,j). Interestingly, *L. murinus* did not exert the same effect, instead supporting an expansion of *Lachnospiraceae* through the course of disease (Figures 3g, 5j, and S2).

The complex interactions among particular constituents of the gut microbiome are of particular import to understanding their combined functional metabolic output and interface with the host. Such interactions can be cooperative or competitive, including the secretion of a wide array of bioactive small molecules, specifically designed to increase fitness of the bacterial species responsible for their production, and notably include small antimicrobial peptides targeting closely related taxa, or so-called bacteriocins. Using *in vitro* microbiome models, we first demonstrated sufficiency of *L. reuteri* to diminish SCFA producer abundance and metabolite production (Figure 6a-h). In search of potential effectors encoded by *L. reuteri* to mediate this effect, we identified several loci encoding antimicrobial peptides within the genome of *L. reuteri*, including enterolysin A, acidocin, and gassericin (Figure 6i), with known activity against SCFA producers.^112–116,122–124^ Of note, gassericin has indistinguishable sequence homology to another *L. reuteri* encoded bacteriocin, reutericin 6, which also displays activity against known SCFA producers.^124^ Interestingly, reutericin 6 biosynthesis and synthesis of other polyketides has been previously linked to fatty-acid metabolism.^125^ The proposed pathway involved in reutericin 6 biosynthesis in *L. reuteri*, is encoded as a genetic island lacking the essential enzymes to process the necessary acyl-chains,^126^ suggesting a potential link between *L. reuteri* depletion of fatty acids as observed in monoculture (Figure 5) and production of antimicrobial peptides targeting SCFA-producing microbiota. Future studies using genetic editing of *L. reuteri* isolates will be needed to confirm a functional requirement for these loci in depleting SCFA producers.

More broadly, the contribution of competitive interactions among the microbiota to altered composition of the gut microbiome in pwMS remains to be fully elucidated. However, outside of a role in CNS autoimmunity, some evidence for bacteriocin driven shifts in the gut microbiota as impacting SCFA-producer abundance does exist. Leveraging an *in vitro* culture of human fecal samples from divergent gut microbiomes, Pu and colleagues identified an enterotype-specific response to treatment with plantaricin NC8, altering the levels of *Bacteroides*, *Bifidobacterium*, *Prevotella*_9, and *Akkermansia* and further diminishing the abundance of *Lachnospiraceae*, correlating with diminished fecal SCFA abundance, similar to our own results driven by *L. reuteri* encoded bacteriocins.^127^ By contrast, plantaricin K, administered in drinking water to chickens, increased the abundance of *Lachnospiraceae*, reminiscent of the effects of *L. murinus*.^128^ Notably, plantaricin NC8, plantaricin K, as well as acidocin and gassericin putatively encoded by *L. reuteri* and sakacin encoded by *L. murinus*, are all class II bacteriocins, underscoring the differential specificity among distinct bacterial produced antimicrobials and their divergent effects on the gut microbiota. Importantly, precisely due to this purported specificity, bacteriocins are touted as potential therapeutics, reshaping the gut microbiome to benefit human health and disease,^123,129^ although their capacity to alter the composition of the human gut microbiota is less clear.^130,131^ These data highlight the necessity to define the precise microbial targets, their metabolic function, and any interactions with the host, to enhance any therapeutic potential and avoid deleterious unanticipated off-target effects.

We have previously shown that exacerbation of EAE by *L. reuteri* involves another major bacterial metabolic pathway: metabolism of dietary tryptophan.^68^ We believe that this occurs through direct metabolism of tryptophan by *L. reuteri* into immunomodulatory metabolites, including indoles and p-cresols. In contrast, the effect of *L. reuteri* on EAE via modulation of SCFA production, as described in the present study, is indirect, via competition with other gut commensals. We hypothesize that these two effects are independent, but not mutually exclusive, and thus likely occur in parallel, contributing additively to the overall effect of *L. reuteri* on EAE in a setting where dietary substrates for both pathways are available. Interestingly, we have previously shown that the genome of our *L. murinus* isolates also encode some of the machinery required for tryptophan metabolism, although with distinct differences in the aromatic amino acid aminotransferase (ArAT) loci and a lack of aliphatic amidase E (amiE) as compared with *L. reuteri*.^68^ Recent studies by Alexander and colleagues suggest that tryptophan metabolism by *L. murinus* may drive protective effects in the EAE model,^132^ which is also somewhat consistent with a lack of EAE exacerbation by *L. murinus* colonization in the present study (Figure 1). However, we also note an important difference in methodology: while we introduced *L. murinus* endogenously by stable colonization and vertical transmission in our study, Alexander and colleagues used continuous gavage with exogenous cultured bacterium in their study. Similarly, previous studies have also reported conflicting findings concerning the role of *L. reuteri* in modulating CNS autoimmunity. Consistent with our own findings, also using stable one-time colonization with a murine commensal isolate of *L. reuteri*, Miyauchi and colleagues found that the gut microbiome context impacts *L. reuteri* driven exacerbation of CNS autoimmunity.^133^ In contrast, studies utilizing daily gavage with high doses of probiotic strains of *L. reuteri*, have shown an anti-inflammatory effect to reduce disease severity.^134,135^ The disparate effects of *L. reuteri* on CNS autoimmunity likely stem from four main factors 1) the strain of *L. reuteri* (probiotic or commensal, with underlying genetic differences likely influencing host–microbe interactions), 2) mode of administration (daily high-dose gavage or stable one-time colonization), 3) duration of exposure (at or just prior to disease induction compared to lifelong commensal colonization) and 4) the gut microbiome community context (dictating microbe–microbe co-operative and competitive interactions as ultimately impacting the host). With regard to the latter, our study highlights the importance of SCFA-producing gut microbiota in *L. reuteri* driven autoimmune disease, underscoring a role for gut microbiome composition in contextual effects of *Lactobacillaceae* on CNS pathogenesis.

Our data highlight the divergent roles of specific commensal *Lactobacillaceae* species in modulating global gut microbiota metabolism and CNS autoimmunity, and point toward the baseline gut microbiome as a modifier of the efficacy of dietary intervention strategies in pwMS. Moreover, we propose a novel mechanism whereby SCFA producers may be depleted in pwMS through competitive interactions between the gut microbiota, suggesting that therapeutic restoration of SCFA levels in pwMS may require not only dietary intervention, but also modulation of the gut microbiome composition.

## Supplementary Material

Supplemental Material

Supplemental Material

Supplemental tables.xlsx

## Data Availability

All data that support the findings of this study are available from the corresponding author upon request. The datasets generated and/or analyzed during the current study are available at the NCBI Sequence Read Archive repository, https://www.ncbi.nlm.nih.gov/sra (BioProject ID PRJNA1106467).
